# Assessment of Gender Equity Among Invited Speakers and Award Recipients at US Annual Medical Education Conferences

**DOI:** 10.1001/jamanetworkopen.2019.16222

**Published:** 2019-11-27

**Authors:** Halah Ibrahim, Sawsan Abdel-Razig, Dora J. Stadler, Joseph Cofrancesco, Sophia Archuleta

**Affiliations:** 1 Sheikh Khalifa Medical City, Abu Dhabi, United Arab Emirates; 2 New York University School of Medicine, New York; 3 Weill Cornell Medicine-Qatar, Doha, Qatar; 4 Johns Hopkins University School of Medicine, Institute for Excellence in Education, Baltimore, Maryland; 5 National University Health System, Singapore, Singapore

## Abstract

This cross-sectional study assesses the gender balance of invited speakers and award recipients at US annual medical education conferences.

## Introduction

Gender inequity in academic medicine persists despite decades of studies highlighting disparities in salary, publications, and academic rank and implementation of initiatives to improve the representation of women.^1^ Gender inequity has also been demonstrated in awards and speaker opportunities at major specialty-specific conferences.^2,3^ As medical education grows as a discipline, medical education conferences are increasing in number and attendance. The Association of American Medical Colleges (AAMC) and the Accreditation Council for Graduate Medical Education (ACGME) annually host the premier conferences focusing on undergraduate and postgraduate medical education in the United States. We examined these conferences to assess gender parity over the past decade in the number of invited speakers and award recipients.

## Methods

In this cross-sectional study, award recipients (AAMC 2009-2018 and ACGME 2010-2019) and invited plenary and keynote speakers (2010-2019) were identified through online searches of conference websites, brochures, and annual reports. Gender was analyzed using the validated online Gender Balance Assessment Tool, which uses the genderize.io algorithm to estimate the probability that a given name belongs to a woman.^4^ Where the result of the Gender Balance Assessment Tool was inconclusive (4 instances of initials and 3 foreign names), we determined gender from photos and pronouns used on conference materials. For speaker data, we excluded session chairs, nonplenary panels, and preconference and postconference sessions. For award data, we excluded groups, posters, and women-only categories. Proportions of women were compared using Fisher exact test (speakers) and χ^2^ test (awardees). Statistical significance was set at 2-tailed *P* < .05. The Johns Hopkins University School of Medicine institutional review board deemed this study exempt as the data analyzed were publicly available. Reporting of this study follows the relevant portions of the Strengthening the Reporting of Observational Studies in Epidemiology (STROBE) reporting guideline.

## Results

In the 10-year period and 20 conferences studied, 12 of 41 invited plenary and keynote speakers (29%) were women (Table 1). This did not significantly differ by conference. Women were underrepresented across all award categories, accounting for 53 of 193 total recipients (27%) (Table 2). Proportions of female awardees were similar in both the AAMC and ACGME conferences (difference, 2.0% [95% CI −12.0% to 14.4%]; *P* = .77). Women were most represented in the AAMC Arnold P. Gould Foundation Humanism in Medicine (4 [40%]) and ACGME Parker J. Palmer Courage to Lead (11 [38%]) awards. They were least represented in the AAMC Alpha Omega Alpha Robert J. Glaser Distinguished Teacher award (8 [20%]).

**Table 1.** Representation of Women Among Plenary and Keynote Speakers at AAMC and ACGME Annual Medical Education Conferences (2010-2019)

Conference Name	Total No. of Speakers	No. of Female Speakers (% of Total)
AAMC	30	10 (33)
ACGME	11	2 (18)

Abbreviations: AAMC, Association of American Medical Colleges; ACGME, Accreditation Council for Graduate Medical Education.

**Table 2.** Representation of Women Among Award Recipients at AAMC (2009-2018) and ACGME (2010-2019) Annual Medical Education Conferences

Conference and Award Name	Total No. of Award Recipients	No. of Female Award Recipients (% of Total)
AAMC		
Abraham Flexner Award for Distinguished Service to Medical Education	12	4 (33)
Alpha Omega Alpha Robert J. Glaser Distinguished Teacher Award	40	8 (20)
Arnold P. Gold Foundation Humanism in Medicine Award	10	4 (40)
All	62	16 (26)
ACGME		
John C. Gineapp Distinguished Service Award^a^	4	1 (25)
Parker J. Palmer Courage to Lead Award	29	11 (38)
Parker J. Palmer Courage to Teach Award	98	25 (26)
All	131	37 (28)

Abbreviations: AAMC, Association of American Medical Colleges; ACGME, Accreditation Council for Graduate Medical Education.

^a^ Not awarded annually.

## Discussion

Based on data from 2 prestigious conferences, significant gender inequity exists in speaking opportunities and recognition of medical educators. Less than one-third of plenary speakers and award recipients were women, while in the corresponding decade, women medical school faculty increased from 36% to 41%.^5^ Our data fit a larger pattern of documented gender differences in speakerships and award presentations at specialty-specific meetings.

The relatively higher representation of women awardees in humanism is not unexpected, as studies confirm that women are more likely to receive awards related to teaching and humanism and less likely to be recognized for distinction in research or leadership.^6^ We are therefore encouraged by the higher percentage of women recipients of the ACGME Courage to Lead award, which may increase mentorship and role modeling opportunities for female trainees. Study limitations include the genderize.io algorithm’s tendency to overestimate the percentage of women and only recognize binary genders.

Medical education conferences afford visibility and serve as important forums to disseminate scholarly work. Presenting and receiving recognition at national meetings are hallmarks of academic success and important factors for promotion of medical educators. As these conferences are attended by learners, leaders, and educators of future generations of physicians, award recipients and key speakerships affect more than just the individual. The public and visual recognition sends a powerful message of what and whom academic medicine considers important. Selection choices can encourage diversity and inclusivity or reinforce a culture of gender inequity in academic medicine as a whole. Increased representation of women in key speakerships and awards at major medical education conferences provides an important opportunity that can be systematically targeted. Medical education can, and should, lead the way in promoting gender equity.
